# Analysis of the Differential Exosomal miRNAs of DC2.4 Dendritic Cells Induced by *Toxoplasma gondii* Infection

**DOI:** 10.3390/ijms20215506

**Published:** 2019-11-05

**Authors:** Dong-Liang Li, Wei-Hao Zou, Sheng-Qun Deng, Hong-Juan Peng

**Affiliations:** Department of Pathogen Biology, Guangdong Provincial Key Laboratory of Tropical Disease Research, School of Public Health, Southern Medical University, Guangzhou 510515, China

**Keywords:** *Toxoplasma gondii*, dendritic cells, exosome, miRNA

## Abstract

*Toxoplasma gondii* is an intracellular parasite that infects humans and other warm-blooded animals. Exosomes are endocytic-derived vesicles released by cells, representing an important mode of intercellular communication. In exosomes, specific molecules of proteins, lipids, and mRNAs or miRNAs have been detected, some of which are capable of transferring biologically active molecules to recipient cells. Dendritic cells (DCs) are the only antigen-presenting cells (APCs) that activate the initial immune response. In this study, high-throughput sequencing was used to analyze the exosomal miRNA profile of DC2.4 cells infected with *Toxoplasma gondii* for 28 h, compared with those of uninfected DC2.4 cells. Differential exosomal miRNAs (DEmiRs) from these two cell groups were analyzed. Through high-throughput sequencing, 3434 DEmiRs were obtained, and 12 stably enriched DEmiRNAs were verified by Reverse Transcription-quantitative Polymerase Chain Reaction (RT-qPCR) and selected for further analysis. The target genes of these 12 miRNAs were predicted with online analysis software and subjected to bioinformatics analyses including protein–protein interaction (PPI) network analysis, key driver analysis (KDA), gene ontology (GO) enrichment, and Kyoto encyclopedia of genes and genomes (KEGG) pathway analysis. These DEmiRs were found to be associated with a variety of biological processes and signaling pathways involved in host ubiquitin system, innate immunity, biosynthesis, and transferase activity and could be potential biomarkers for *T. gondii* infection.

## 1. Introduction

*Toxoplasma gondii* is an intracellular parasite that infects humans and other warm-blooded animals. It is estimated that one-third of the world’s population is seropositive with this parasitic infection [1]. It usually results in asymptomatic latent infection in immune competent hosts and leads to devastating diseases in immunodeficient individuals and fetuses/newborns through congenital transmission in the primary infection during pregnancy [2,3]. *Toxoplasma* tachyzoites can hide in white blood cells and reach all parts of the body through the bloodstream, resulting in suppressive immunity and various diseases [4].

Exosomes are endocytic-derived vesicles released by cells, which represent an important mode of intercellular communication by acting as a transporter between the membrane and the cytosolic contents [5] and facilitate processes such as antigen presentation [3]. In exosomes, specific molecules of proteins, lipids, and mRNAs or miRNAs have been detected, and some exosomes are capable of transferring biologically active molecules to recipient cells [6,7]. Exosomes play a key role in cell-to-cell communication [4], and studies have shown that exosomes can function in parasite–host interactions [8].

Dendritic cells (DCs) are the most important professional antigen-presenting cells (APCs) in the human body and are the only APCs that activate the initial immune response. As APCs, dendritic cells are promoters and regulators of the immune system, which can effectively activate B cells and T cells [9]. It has been reported that DC-derived exosomes can be used for immunoprophylaxis against *T. gondii* and other pathogens [10].

As a member of the non-coding RNA family, miRNAs are endogenous RNAs of 19–23 nucleotides in length, which can be specifically identified [11]. They target mRNAs to regulate the gene expression at the post-transcriptional level and are involved in the regulation of cell proliferation, differentiation, and apoptosis, as well as in the intercellular signaling and communication [11]. Many miRNAs have been identified as biomarkers for some diseases [12].

In this study, high-throughput sequencing was used to analyze and compare the exosomal miRNA profiles of the DC2.4 cells infected with *T. gondii* and the uninfected DC2.4 cells. The stably enriched differential exosomal miRNAs (DEmiR) from these two cell groups were further analyzed. The target gene prediction for these miRNAs provides us with hints to further explain the molecular mechanism of DC cells reacting to *T. gondii* infection.

## 2. Results

### 2.1. Evaluation of the Efficiency of T. gondii RH Tachyzoites Invading DC2.4 Cells

**Figure 1:**
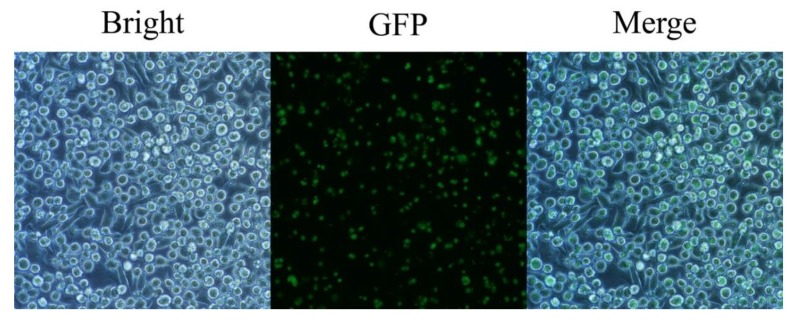
Detection of the efficiency of *Toxoplasma gondii* GFP labeled RH (RH-GFP) tachyzoites invading DC2.4 cells (4000×). The DC 2.4 cells were infected with GFP-labeled RH tachyzoites of *T. gondii* (multiplicity of infection, MOI = 3). It was observed that each of the DC2.4 cells was infected under a fluorescence microscope with a GFP filter.

Considering that DC2.4 cells are mouse-marrow-derived antigen presenting cells (APCs), possibly there is an antagonistic effect on *T. gondii* invasion. Therefore, we detected the infection rate of DC2.4 cells by *T. gondii* using Green Fluorescence Protein (GFP)-labeled RH tachyzoites in infection. The infection was performed at a multiplicity of infection of three (MOI = 3), the invasion of the fluorescent tachyzoites in the DC2.4 cells were visualized under a fluorescence microscope with GFP filter (Figure 1). Basically, each of the cells had been invaded by a GFP-RH tachyzoite, indicating that *T. gondii* RH strain can infect DC2.4 cells efficiently, and this cell model can be used for our next experiments.

### 2.2. Qualification and Quantification of the Exosomes

**Figure 2:**
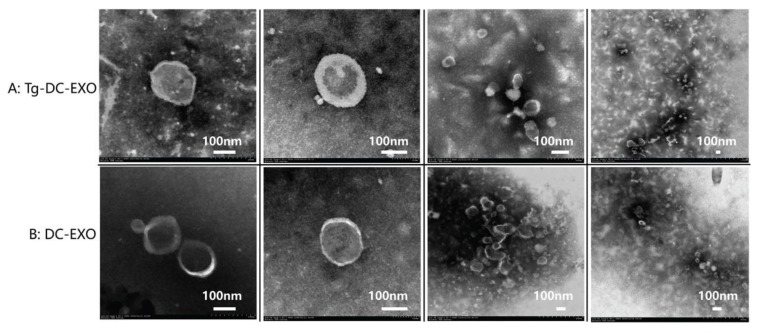
Detection of exosomal extracts with transmission electron microscope (TEM). The exosomes were visualized under a transmission electronic microscope (Hitachi, Northeastern Honshu, Japan) at 80 kV voltage, with a magnification of 10K–30K. (**A**) Exosomes extracted from the DC2.4 cells infected with *T. gondii* RH tachyzoites (MOI = 3) for 28 h. (**B**) Exosomes extracted from DC2.4 cells after 28 h culturing. Obviously, the tea-tray-like structure was observed under a TEM.

**Figure 3:**
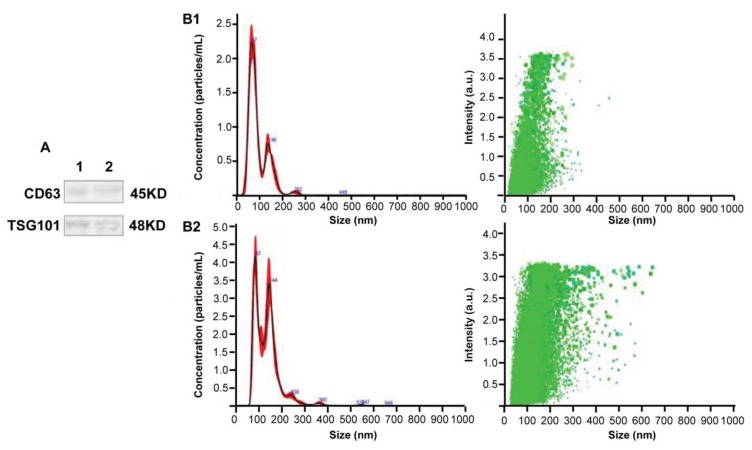
Identification of exosomal extracts with western blot and nanoparticle tracking analysis (NTA). (**A**) The exosome-specific proteins Tumor Sensitive Gene 101 (TSG101) and Lysosomal associated membrane protein 3 (CD63) were detected in the exosomal extracts of both DC2.4 cells uninfected and infected with *T. gondii*. (**B1**,**B2**) NTA results showed that the size of the vesicles peaked at an average of 50–200 nm in diameter.

The DC2.4 cells were cultured with exosome-free Roswell Park Memorial Institute (RPMI) 1640 complete medium, and the exosomes were extracted from the culture of approximately 7.52 × 10^8^ DC cells infected with *T. gondii* RH strain or uninfected. In Group A (*Tg*-DC-EXO), exosomes were extracted from the culture of DC2.4 cells infected with RH strain (MOI = 3) at 28 h post infection; and in Group B (DC-EXO), exosomes were extracted from the DC2.4 cells cultured for 28 h without infection. The transmission electron microscope (TEM) analysis indicated that these vesicles had a complete continuous bilayer membrane with a diameter of approximately 100 nm (Figure 2). In addition, identification of the exosomes was performed by detecting the exosomal-specific proteins Tumor Sensitive Gene 101 (TSG101) and Lysosomal associated membrane protein 3 (CD63) with western blotting (Figure 3). Furthermore, nanoparticle tracking analysis (NTA) was used to determine the size distribution of the vesicles, and we found the average diameter reaching a peak at 50–200 nm, which is a typical morphological feature of the exosomes and is consistent with the size of exosomes extracted from *T. gondii* [13] and the other cells [14] (Figure 3).

### 2.3. miRNA Sequencing and Analysis of the Differential Exosomal miRNAs (DEmiRs) from the DC2.4 Cells Uninfected and Infected with T.gondii RH Tachyzoites

High-throughput sequencing results were used to compare the exosomal miRNA profile between group A (DC2.4 cells infected with RH tachyzoites) and group B (uninfected DC2.4 cells). As the experiment was performed in biological triplicates for each group and repeated three times, nine sets of data of DEmiRs were generated, including A1/B1, A1/B2, A1/B3 (Supplementary Table S1), A2/B1, A2/B2, A2/B3 (Supplementary Table S2), and A3/B1, A3/B2, A3/B3 (Supplementary Table S3). By screening with the threshold of log2 (A/B) ≥1 and false discovery rate (FDR) ≤0.05, a total of 3434 DEmiRs were obtained after combining the nine sets of data. Finally, 12 stably enriched DEmiRs (which were found in all of the nine sets of data) were screened from the nine sets of comparisons, which are shown in Table 1.

### 2.4. Verification of the Stably Enriched DEmiRs with RT-qPCR

**Figure 4:**
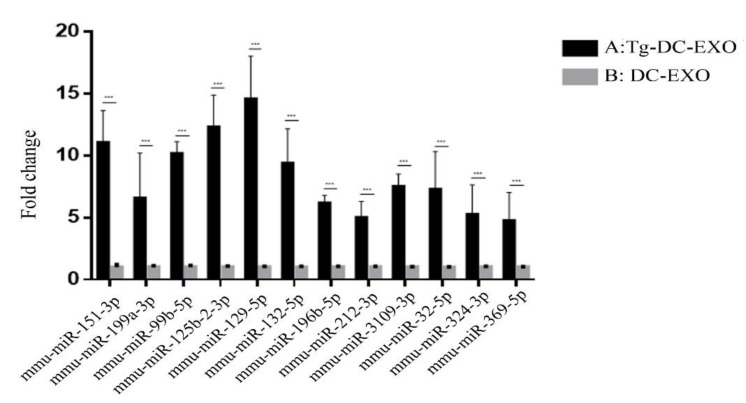
Quantititive Polymerae Chain Reaction (qPCR) verification of the enrichment level of the stably enriched differential exosomal miRNAs (DEmiRs). Stem-loop Reverse Transcription- quantititive Polymerase Chain Reaction (RT-qPCR) was performed to compare the relative enrichment level of the candidate miRNAs normalized with the internal control U6 small nucleic RNA (snRNA) level, between the exosomes from group A (*Tg*-DC-EXO) and group B (DC-EXO). The enrichment level of each DEmiR from group B (the grey column) was set as 1. The height of the black columns represented the folds of the enrichment level of the indicated miRNAs from group A being divided by the level form group B. The p-value was calculated by the SPSS software. *** *p*-value < 0.005. X: miRNA ID. Y: Fold change.

Regarding the 12 stably enriched DEmiRs, the enrichment level was verified by using RT-qPCR. The reverse transcription primers and qPCR primers are shown in Table 2. The RT-qPCR results are shown in Figure 4, which shows that the difference of the enrichment level of these 12 miRNAs between the infected and uninfected DC2.4 cells was consistent with the high-throughput sequencing result.

U6 small nucleic RNA (snRNA) was used as an internal reference. In reverse transcription, miRNAs were reverse transcribed using stem-loop primers, and U6 was reverse transcribed with U6 R. In qPCR, U6 was amplified with the U6 F and U6 R primers, miRNAs were amplified with the specific forward primers and the common stem-loop downstream primer (Universal Reverse Primer, URP).

### 2.5. Prediction and Analysis of the Target Genes of the Stably Enriched DEmiRNAs

**Figure 5:**
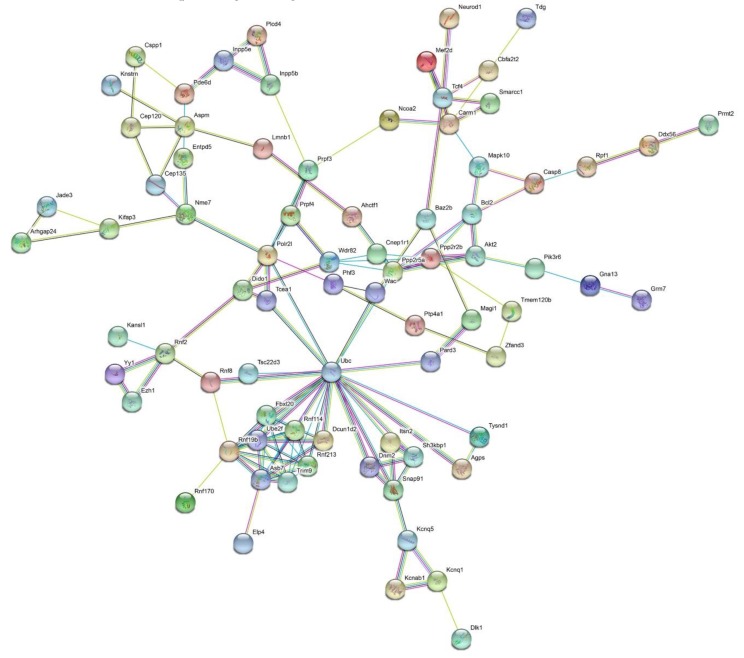
Protein–protein interaction analysis for the proteins coded by the target genes of the 12 stably enriched DEmiRs. Proteins with less than five associated proteins were deleted from the network (PPI enrichment, *p* < 1 × 10^−16^). Number of nodes: 78; number of edges: 138; average node degree: 3.54; average local clustering coefficient: 0.506; expected number of edges: 58. The PPI network showed that Polyubiquitin-C (UBC) was the most prominent (primary) hub with 14 related proteins, and then Fbx120, Rnf 114, Dcun1d2, Rnf 213, Trim9, Asb7, Rnf 19b, UBE2f, and Aspm were the secondary hubs with six associated proteins for each.

Target gene prediction was performed for the 12 stably enriched DEmiRs with TargetScan and MiRanda software, and 284 target genes were predicted with both. These target genes were further subjected to protein–protein interaction (PPI) analysis. The PPI network (*p* < 1 × 10^−16^) shows that Polyubiquitin-C (UBC) is the most prominent (primary) hub with 14 related proteins, and then Fbx120, Rnf 114, Dcun1d2, Rnf 213, Trim9, Asb7, Rnf 19b, UBE2f, and Aspm are the secondary hubs with six associated proteins for each. Most of these hub proteins are related to ubiquitin connexin. For example, UBE2f reduces characteristic neddylation E2 and regulates cell viability; Rnf 19b is associated with E3 ubiquitin ligase (Figure 5).

**Figure 6:**
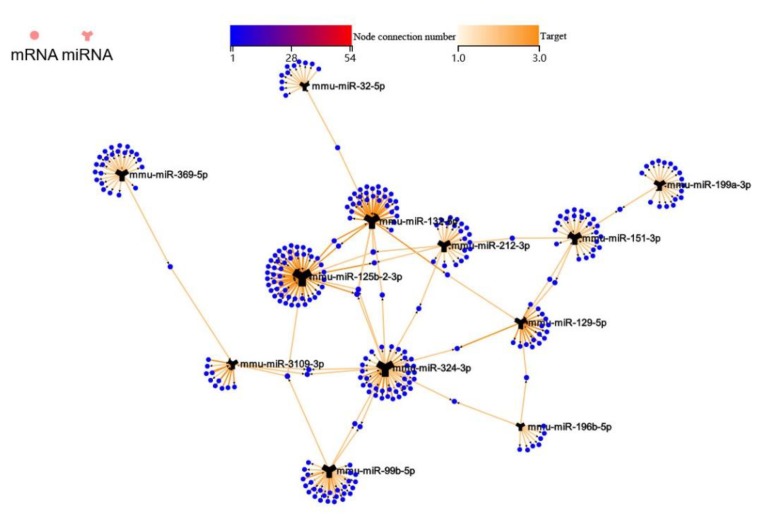
The correlation between the 12 stably enriched DEmiRs and their target mRNAs. The association network of these DEmiRNAs and their target mRNAs, indicated that miR-132-5p, miR-324-3p, and miR-125b-2-3p were the most correlated miRNAs sharing two common target genes with each other.

In order to understand how these stably enriched DEmiRs potentially function on their predicted target mRNAs, the association network of these DEmiRNAs and their target mRNAs was drawn (Figure 6). The result indicated that miR-132-5p, miR-324-3p, and miR-125b-2-3p were the most correlated miRNAs sharing two common target genes with each other (Figure 6).

**Figure 7:**
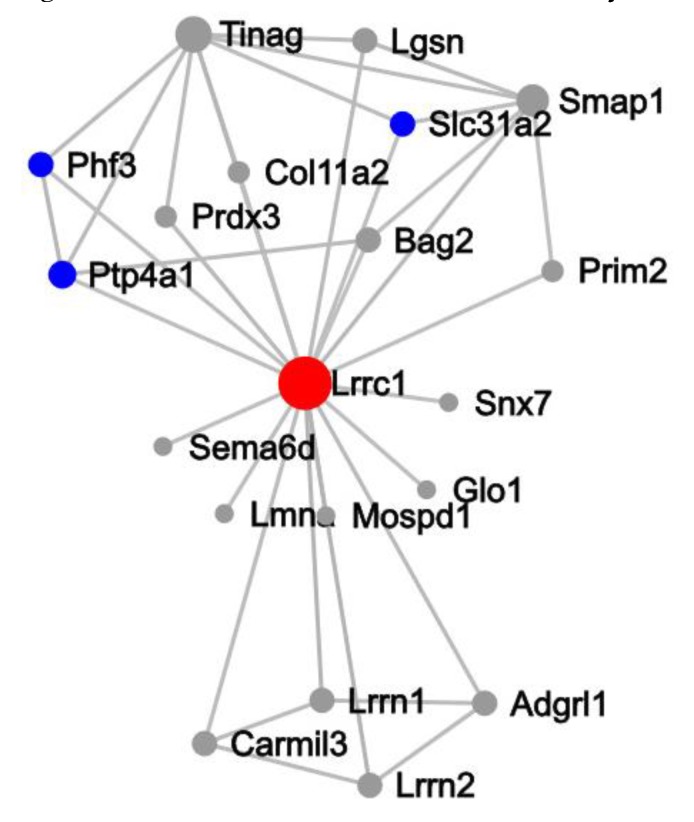
Key driver analysis (KDA) for the predicted target genes of the stably enriched DEmiRs. The result showed that *Lrrc1* was the most prominent (primary) hub among the target genes, Tnag and Smap1 were the secondary hubs, and *Ptp4a1*, *Phf3*, *Lgsn*, *Slc31a2*, *Bag2*, *Lrrn1*, *Adgrl1*, *Lrrn2*, and *Camil3* were the tertiary hubs.

Furthermore, in order to clarify the roles of these 284 predicted target genes, they were subjected to key driver analysis (KDA). It showed that leucine rich repeat 1 (*Lrrc1*) was the most prominent (primary) hub among these target genes, Tnag and Smap1 were the secondary hubs, and *Ptp4a1*, *Phf3*, *Lgsn*, *Slc31a2*, *Bag2*, *Lrrn1*, *Adgrl1*, *Lrrn2*, and *Camil3* were the tertiary hubs (Figure 7).

**Figure 8:**
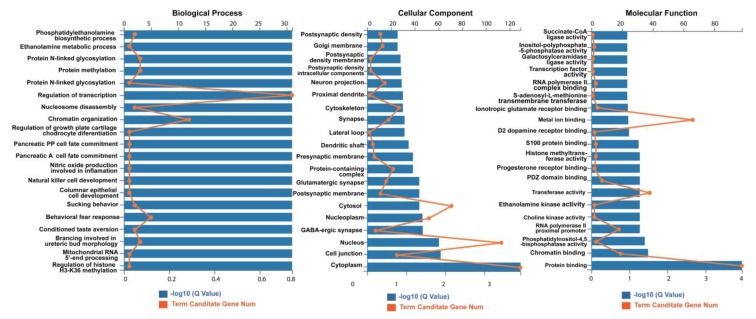
The gene ontology (GO) term enrichment for the predicted target genes of the 12 stably enriched DEmiRs. The result indicated that these target genes were involved in “regulation of growth plate cartilage chondrocyte differentiation” (Q = 0.1995) and “chromatin organization” (Q = 0.5012) in biological process; “cytosol” (Q = 0.01), “nucleus” (Q = 0.0003), and “cytoplasm” (Q = 0.0001) in cellular component; “metal ion binding” (Q = 0.0032), “transferase activity” (Q = 0.0316), and “protein binding” (Q = 0.0001) in molecular function (significance value, Q < 0.05).

The gene ontology (GO) enrichment for these target genes revealed that the 12 stably enriched DEmiRs were significantly involved in “regulation of growth plate cartilage chondrocyte differentiation” (Q = 0.1995) and “chromatin organization” (Q = 0.5012) in biological process; “cytosol” (Q = 0.01), “nucleus” (Q = 0.0003), and “cytoplasm” (Q = 0.0001) in cellular component; and “metal ion binding” (Q = 0.0032), “transferase activity” (Q = 0.0316), and “protein binding” (Q = 0.0001) in molecular function (Figure 8). The significance value was set at Q < 0.05. The enrichment in biological process is not significant, while it is significant in cellular component and molecular function.

**Figure 9:**
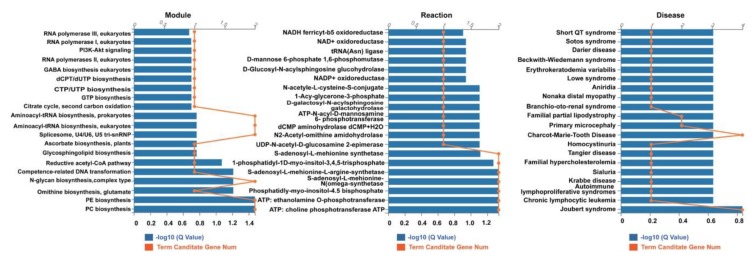
The Kyoto Encyclopedia of Genes and Genomes (KEGG) pathway aggregation for the target genes of the 12 stably enriched DEmiRs. The result indicated that these target genes were involved in “aminoacyl-RNA biosynthesis in prokaryotes and eukaryotes, spliceosome, U4/U6, U5 tri-snRNP, N-glycan biosynthesis, complex type, phosphatidylethanolamine (PE) biosynthesis, and phosphatidylcholine (PC) biosynthesis (Q = 0.03) in module group; “*S*-adenosyl-l-methionine, phosphatidly-myo-inositol-3.4.5 bisphosphate, ATP: ethanolamine O-phosphotransferase, ATP: choline phosphotransferase ATP” (Q = 0.05) in reaction group; and Charcot–Marie–Tooth disease and Joubert syndrome” (Q = 0.16) in disease group.

**Figure 10:**
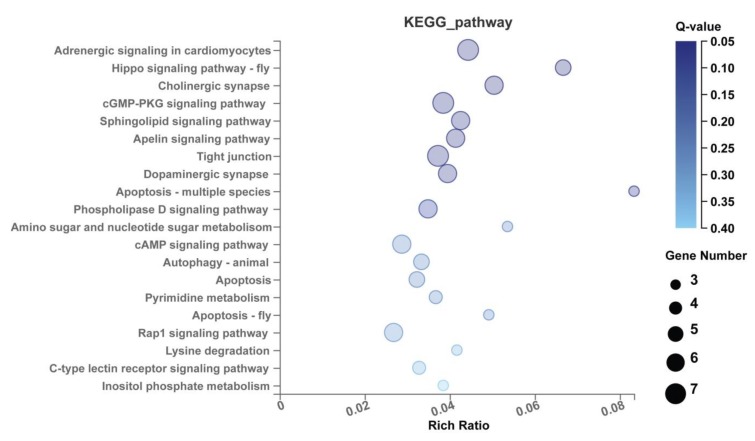
A bubble result graph of KEGG aggregation for the target genes of the 12 stably enriched DEmiRs. It is clear that the 12 stably enriched DEmiRs significantly functioned in “adrenergic signaling in cardiomyocytes (Q = 0.05).

Kyoto Encyclopedia of Genes and Genomes (KEGG) pathway aggregation for these target genes revealed that the 12 stably enriched DEmiRs significantly functioned in “aminoacyl-tRNA biosynthesis in prokaryotes and eukaryotes, spliceosome, U4/U6, U5 tri-snRNP, N-glycan biosynthesis, complex type, phosphatidylethanolamine (PE) biosynthesis, and phosphatidylcholine (PC) biosynthesis (Q = 0.03) in module group; “S-adenosyl-L-methionine, phosphatidly-myo-inositol-4.5 bisphosphate, Adenosine Triphosphate (ATP): ethanolamine O-phosphotransferase, ATP: choline phosphotransferase” (Q = 0.05) in reaction group; and Charcot–Marie–Tooth disease and Joubert syndrome” (Q = 0.16) in disease group (Figure 9). In particular, they seem to function in “adrenergic signaling in cardiomyocytes” (Q = 0.05) (Figure 10).

## 3. Discussion

Dendritic cells are the most potent antigen-presenting cells in the immune system and they determine the adaptive immune responses during infection [15]. In our study, we verified that *T. gondii* RH tachyzoites could infect DC2.4 cells efficiently and resulted in a differential exosomal miRNA profile after infection. As a nanoscale membranous structure secreted by host cells, exosomes carry a variety of biological functional factors, including miRNA, and the differential miRNAs enriched in the exosomes indirectly reflect the changes of miRNAs in the host cells [16]. As the source of DC cells, colloidal cells transport the bioactive substances to T cells through membrane fusion or endocytosis, and thereby regulate T cells’ function. In this way, the exosomes transmit information among cells and affect the process of the immune system [1]. Through their regulation on mRNA degradation, interference with protein synthesis, or inhibition of protein translation, miRNAs regulate cell apoptosis, differentiation, proliferation, and growth [17]. The results of high-throughput sequencing indicated that the invasion of DC2.4 cells by *T. gondii* resulted in significant changes in exosomal miRNA profile, and the enriched differential exosomal miRNAs (DEmiRs) may represent the miRNA differences in their host cells.

In this study, 12 miRNAs were identified as stably enriched DEmiRs through comparison of the exosomal miRNAs from the uninfected DC2.4 cells and those infected with *T. gondii*, and further verified by RT-qPCR which showed consistent results with the high-throughput miRNA sequencing (Figure 4). Among the 12 stably enriched DEmiRs, upregulation of miR-196b-5p has been reported to maintain tumor stem cell activity and promote colorectal cancer cell resistance by activating the signal transducer and activator of transcription (STAT3) signaling pathway; and to regulate the migration and metastasis of colorectal cancer cells through Homeobox protein Hox-B7 (HOXB7) and polypeptide N-acetylgalactosaminyltransferase 5 (GALNT5) interaction [18]. Meanwhile, upregulation of miR-196b-5p is also reported to promote the proliferation and invasion of gastric cancer cells by targeting STAT3 pathway, and miR-196b-5p in macrophages is regulated by DNA methylation in the promoter region of *hoxa10* [19,20]. miR-125b-2-3p can be used as a biomarker for ischemic stroke [21]. miR-132-5p is related to the improved recombinant protein production in expression cells [22]. miR-324-3p regulates cell growth and apoptosis by targeting Mothers Against DPP Homolog 7 (SMAD7), and inhibits the migration and invasion of nasopharyngeal carcinoma by targeting wingless-type MMTV integration site 2B (WNT2B) [23,24]. miR-369-5p is associated with tumor migration [25], whereas miR-151-3p inhibits lipopolysaccharide (LPS)-induced IL-6 production by targeting STAT3 [26,27]. miR-199a-3p has been shown to be associated with liver cancer and pathological changes in the liver and to be a potential biomarker for the diagnosis of hepatocellular carcinoma [28,29]. miR-99b-5p is associated with gastric cancer and involved in the regulation of the immune system [30], and may serve as a biomarker for communication in the immune system [31]. miR-212-3p is related to cancer and the immune system [32], and miR-3109-3p is involved in gastric cancer [33].

The dendritic cells themselves are considered to have a central role in the immune system. The comparison of the exosomal components from the DC2.4 infected or uninfected with *T. gondii* may reflect the regulation of *T. gondii* on the host cell immunity, as well as the regulatory mechanisms. It has been reported that *T. gondii* infection inhibits tumor growth in the Lewis lung carcinoma mouse model through the induction of *Th1* immune responses and anti-angiogenic activity [34]. Dendritic cells are antigen-presenting cells and have strong links with diseases and the immune system. Exosomes secreted by dendritic cells after *Toxoplasma* infection may have a great impact on the regulation of the immune system.

These 12 stably enriched DEmiRs were subjected to target gene prediction with miRanda and TargetScan online software. Two hundred and eighty-four target genes were obtained for subsequent bioinformatics analysis, and as a result, the immune-related genes and signaling pathways were specifically concentrated. Firstly, protein–protein interaction (PPI) analysis for the 284 target genes showed that the hub proteins coded by the targets gene were mostly ubiquitin system. For example, the *ubc* gene coded a polyubiquitin precursor with exact head-to-tail repeats, and the number of the repeats differed between species and strains; UBE2f reduced characteristic neddylation E2 and regulated cell viability; and RNF19 was associated with E3 ubiquitin ligase (Figure 5).

In order to understand how these stably enriched DEmiRs potentially function on their target mRNAs, the association network of these miRNAs and mRNAs was drawn. miR-132-5p, miR-324-3p, and miR-125b-2-3p were shown as the most correlated miRNAs sharing two common target genes with each other (Figure 6).

Furthermore, in order to clarify the roles of these 284 predicted target genes, they were subjected to key driver analysis (KDA). It was found that leucine rich repeat 1 (*Lrrc1*) was the most prominent (primary) hub among these target genes, *Tnag* and *Smap1* were the secondary hubs, and *Ptp4a1*, *Phf3*, *Lgsn*, *Slc31a2*, *Bag2*, *Lrrn1*, *Adgrl1*, *Lrrn2*, and *Camil3* were the tertiary hubs (Figure 7). leucine rich repeat (LRR) motif exists in many immune receptors of animals and plants, and function in the signal transduction and host defense of plants and animals by protein–protein interaction [35,36]. In human, 375 LRRC proteins are predicted by genome-wide bioinformatics analysis and function in innate immunity, and almost 50% of them contain only LRR motifs [37].

GO term enrichment for the target genes showed that they were significantly enriched in transferase activity, and protein binding functions (Figure 8). Kyoto encyclopedia of genes and genomes (KEGG) pathway analysis for these target genes revealed that the 12 miRNAs may function in the biosynthesis signaling pathway of aminoacyl-tRNA, N-glycan, phosphatidylethanolamine (PE), phosphatidylcholine (PC); and metabolism reaction signal pathway of ATP-related transferases (Figure 9), as well as the immune signaling pathway, especially in adrenergic signaling in cardiomyocytes (Figure 10).

In conclusion, these 12 stably enriched DEmiRs were involved in the ubiquitin system, innate immune reaction, transferase activity, protein binding, biosynthesis, and ATP-related signaling pathway. On the other hand, due to the great potential for exosomal miRNAs to be used as biomarkers, if these 12 miRNAs could be used as biomarkers for *T. gondii* infection or prognosis needs further studies urgently.

## 4. Materials and Methods

### 4.1. Parasites and Cell Lines

The *T. gondii* RH and the fluorescent RH-GFP strains were maintained by serial passage in Human Forskin Fibroblast cells (HFFs). The DC2.4 and HFF cell lines were purchased from the American Type Culture Collection company (Manassas, VA, USA). Parasites and cells were all cultured with Dulbecco’s Modified Eagle’s Medium (DMEM) (Gibco) supplemented with 10% fetal bovine serum (FBS) (Gibco) and 1% penicillin/streptomycin (Gibco) at 37 °C and 5% CO_2_. The efficiency of *T. gondii* invading DC2.4 was evaluated by infecting DC2.4 with the RH-GFP strain at a multiplicity of infection (MOI) of three. The intracellular tachyzoites were visualized and photos were taken under a fluorescence microscope (Nikon eclipse Ni) with a GFP filter, at a magnification of 10 × 400.

### 4.2. Exosome Isolation

Extracellular vesicle (EV)-depleted FBS was obtained by overnight ultracentrifugation of FBS at 100,000× *g*. The RPMI 1640 medium (Gibco, Grand Island, NY, USA) supplemented with 10% EV-depleted FBS was used for DC2.4 culturing. The DC2.4 cells were cultured in eight T-75 flasks to 90% confluent, then the cells were washed with Phosphate Buffer Saline (PBS) for three times, and further cultured in EV-depleted RPMI 1640 complete medium. Four flasks of DC2.4 cells were infected with *T. gondii* RH tachyzoites (MOI = 3) for 1 h, then the unrecruited tachyzoites were washed off with PBS three times. The cell groups infected by *T. gondii* and the other four flasks of DC2.4 cells used for experimental control were cultured with EV-depleted RPMI 1640 complete medium for 28 h. The supernatant of the cell culture was collected for exosome isolation. Exosomes were isolated and concentrated by differential ultracentrifugation following the previously reported protocol [2]. To remove the cells and cellular debris, the supernatants were transferred to 50 mL centrifugation tubes and centrifuged at 2000× *g* at 4 °C for 20 min, and then the precipitates were resuspended in PBS and subjected to centrifugation (10,000× *g* for 30 min at 4 °C). The supernatant was filtrated with a 0.22 µm filter and then ultracentrifuged at 100,000× *g* for 1 h at 4 °C. The pellet was resuspended in 1 mL PBS and ultracentrifuged again at 100,000× *g* for 1 h at 4 °C to collect the exosomes. The precipitate containing exosomes was resuspended in 100 µL PBS and filtrated with a 0.22 µm filter and kept at −80 °C before being sent for miRNA sequencing. We repeated this experiment three times, and the exosomes harvested from each group were ready for total RNA extraction and small RNA sequencing.

### 4.3. Detection of the Quality of Exosomes with a Transmission Electron Microscope (TEM)

Firstly, a drop of sample was dropped on the copper mesh, and 2 min later, the excess liquid was wiped off from the edge of the copper mesh with filter paper. Secondly, phosphotungstic acid (3%, pH 7.0) was added drop by drop to the copper mesh, and 2 min later, the excess liquid was wiped off from the edge of the copper mesh with a filter paper. At last, pure water was dropped onto the copper mesh, and then the excess water was wiped off from the edge of the copper mesh with a filter paper. After being air-dried, the copper mesh was ready for detection of the exosomes under a transmission electronic microscope (Hitachi, Northeastern Honshu, Japan), at 80 kV voltage, with a magnification of 10k–30k.

### 4.4. Detection of the Size and Quantity of Exosomes by Nanoparticle Tracking Analysis (NTA)

NTA measurements were performed using a NanoSight NS500 instrument (NanoSight, Malvern Instruments Ltd., Malvern, UK). The NTA is based on a technique for direct detection and visualization of the individual nanoparticles in the suspension, which provides information about the size, concentration, and distribution of the particles. Particles are tracked through the light scattering from a laser source and the paths calculated over time to determine their velocity due to the Brownian motion. Exosomes were diluted with PBS and disaggregated using a syringe and needle (29 gauge). The sample was then injected into a NanoSight sample cubicle. The size of exosomes was measured, and the number of particles per milliliter was calculated as mean ± SD.

### 4.5. Verification of Exosomal Proteins by Western Blotting

The exosomes were lysed with the lysis buffer (Beyotime, Shanghai, China) and about 100 µg total proteins were loaded for SDS-PAGE, and then the proteins were transferred to a polyvinylidene fluoride (PVDF) membrane (Millipore, Billerica, MA, USA). The membrane was firstly incubated in the blocking buffer (20 mM Tris-base, 137 mM NaCl, pH 7.6) containing 5% Bovine Serum Albumin (BSA) and 0.05% Tween 20 (Sigma-Aldrich, St. Louis, USA)) for 2 h at room temperature. After blocking, the membrane was then probed with the rabbit polyclonal CD63 (Abcam, MA, USA) or TSG101 antibody (Abcam, MA, USA) diluted in TBST buffer overnight with gentle shaking at 4 °C. After washing three times with TBST buffer, blots were incubated for 2 h at room temperature with the secondary antibody, peroxidase-conjugated goat anti-rabbit IgG (Santa Cruz, CA, USA). Visualization of the protein bands was performed by incubating the blots with SuperSignal West Pico Chemiluminescent Substrate (Thermo Fisher Scientific, Wilmington, DE, USA) and then documented using the ChemiDoc™ MP System (Bio-Rad, Hercules, USA).

### 4.6. Construction of Small RNA Library, Sequencing of the miRNA, and Bioinformatics Analysis

The total RNA was extracted from the exosomes harvested from each group. Small RNAs of 18–30 nt RNA were separated by PAGE gel. A 5-adenylated, 3-blocked single-stranded DNA adapter was linked to the 3′ end of the selected small RNA. The reverse transcription (RT) primers were then added into the solution and cross-linked to the 3′ adapter of RNAs and excessive free 3′ adapter, followed by adding the 5′ adaptor to be linked to the 5′ end of the products. RT enzyme was added to reverse transcribe the small RNAs into complementary DNAs (cDNAs). The cDNAs with both 3′ and 5′ adaptors were amplified with high-fidelity polymerase. The PCR product of 100~120 bp were purified by PAGE gel to eliminate primer dimer and other byproducts and run for sequencing.

The low-quality reads, adaptors, and other contaminants were eliminated to get clean reads. The length distribution of the clean tags, and common and specific sequences between samples were summarized. The novel miRNAs were predicted and the known miRNAs were annotated.

### 4.7. The Screening of the Differentially Enriched miRNAs

Each RNA sequence is uniformly random from its own sample and sequenced. Therefore, it’s abundance obeys binomial distribution or Poisson distribution. Firstly, we evaluate the binomial distribution, and the *p*-value of each miRNA can be corrected by multiple hypothesis tests using Q-value. Considering the background noise of traditional RNA-seq technology is very low, and the Poisson model coincides with the data very well. We made multiple hypothesis test corrections for *p*-value of the difference test, and control false discovery rate (FDR) to determine the threshold of *p*-value. Those miRNAs with FDR ≤ 0.005 and the absolute value of log2 ratio ≥ 1 are considered as significantly regulated miRNAs. The serial experiments of DC2.4 cells infection by *T. gondii* tachyzoites (MOI = 3), exosomes isolation, total RNA extraction, and small RNA sequencing were repeated three times, and nine sets of data (A1/B1, A1/B2, A1/B3; A2/B1, A2/B2, A2/B3; and A3/B1, A3/B2, A3/B3) were generated. Stably enriched DEmiRs were obtained after combining the nine sets of data. Finally, the stable differential RNAs were screened from all of the nine sets of data.

### 4.8. Verification of the Differential miRNAs with RT-qPCR

Total RNA was isolated from exosomes using an exoRNeasy Serum/Plasma Midi Kit (QIAGEN, Duesseldorf, GER) following the manufacturer’s instructions. The RNA was then reverse transcribed using SuperScript™ II Reverse Transcriptase (Thermo Fisher Scientific, Wilmington, DE, USA) and then amplified using QuantStudio™ real-time PCR system (Thermo Fisher Scientific, Wilmington, DE, USA) with Hieff^®^ qPCR SYBR^®^ Green Master Mix (Yeasen, Shanghai, CHA), and primers (shown in Table 1). The PCR cycling condition comprised an initial denaturation at 95 °C for 30 s followed by 35 cycles of amplification consisting of a 15 s denaturation at 95 °C and a 30 s extension at 60 °C. The enrichment level of miRNAs was normalized against U6 snRNA level, and finally expressed as fold change compared with the level of the miRNA from group B (DC-EXO). The experiments were repeated three times. The fold change differences between the *Tg*-DC-EXO and DC-EXO groups were detected by paired t-test with SPSS.

### 4.9. Bioinformatics Analysis of the Differential Enriched miRNA Target Genes

The target genes of the miRNAs were predicted by inputting the sequences of the miRNAs into the online software MiRanda (http://mirdb.org/) and TargetScan (http://www.targetscan.org/mmu_61/). The relationship of the interaction, co-expression, and regulation among these target genes was further predicted by PPI analysis with Cytoscape version 3.7.1 (https://cytoscape.org/). The interaction network of these miRNAs and mRNAs, and the key driver genes among the predicted target genes, were analyzed with Dr. Tom online software developed by Beijing Genomic Institute (BGI) (http://report.bgi.com/). The predicted target genes were then subjected to Gene Ontology (GO) (http://www.geneontology.org/) enrichment for molecular function, biological process, and cellular component. The hypergeometric distribution method was adopted, and a threshold of *p* < 0.05 was considered significant. Meanwhile, the Kyoto Encyclopedia of Genes and Genomes (KEGG) (https://www.genome.jp/kegg/) analysis was performed for the predicted target genes. The significance of target gene distribution enrichment was analyzed by Fisher’s exact test, and *p* < 0.05 was considered statistically significant. A bubble graph of the KEGG aggregation was drawn with Dr. Tom online software developed by BGI (http://report.bgi.com/).

## 5. Conclusions

Exosomes are endocytic-origin membranous vesicles released by various cells into the extracellular space and function as a mode of intercellular communication. *T. gondii* is an intracellular parasite also producing its own exosomes which can be secreted through the host cells. Dendritic cells (DCs) are the only antigen-presenting cells (APC) that activate the initial immune response, and the exosomes of DCs may function as an important immune signal carrier between immune cells or the other cells. In this study, high-throughput sequencing was used to analyze the exosomal miRNA enrichment profiles of DC2.4 cells infected with *T. gondii* for 28 h or uninfected. Differential exosomal miRNAs (DEmiRs) from these two cell groups were analyzed. In total, 3434 DEmiRs were obtained, and 12 stably enriched DEmiRs were subjected to target gene prediction. The 284 predicted target genes were further analyzed with protein–protein interaction (PPI), key driver analysis (KDA), gene ontology (GO) enrichment, and Kyoto Encyclopedia of Genes and Genomes (KEGG) pathway aggregation.

Taken together, all these analyses indicated the 12 stably enriched DEmiRs were involved in ubiquitin system and host innate immunity, transferase activity, protein-binding, biosynthesis, ATP-related transferases, and adrenergic signaling in cardiomyocytes. These DEmiRs might have great potential to be used as biomarkers for *T. gondii* infection or prognosis, and it is important and meaningful to be further studied.

## Figures and Tables

**Table 1:** Information of the stably enriched differential exosomal miRNAs.

miRNA ID	Sequence
mmu-miR-151-3p	CUAGACUGAGGCUCCUUGAGG
mmu-miR-199a-3p	ACAGUAGUCUGCACAUUGGUUA
mmu-miR-99b-5p	CACCCGUAGAACCGACCUUGCG
mmu-miR-125b-2-3p	ACAAGUCAGGUUCUUGGGACCU
mmu-miR-129-5p	CUUUUUGCGGUCUGGGCUUGC
mmu-miR-132-5p	AACCGUGGCUUUCGAUUGUUAC
mmu-miR-196b-5p	UAGGUAGUUUCCUGUUGUUGGG
mmu-miR-212-3p	UAACAGUCUCCAGUCACGGCCA
mmu-miR-3109-3p	UAGGGCCAUCUCAUCCAGAUA
mmu-miR-32-5p	UAUUGCACAUUACUAAGUUGCA
mmu-miR-324-3p	CCACUGCCCCAGGUGCUGCU
mmu-miR-369-5p	AGAUCGACCGUGUUAUAUUCGC

**Table 2:** Reverse transcription primers and qPCR primers for validation of the DEmiRs.

miRNA ID	Reverse Transcription Primer (5′ to 3′)	qPCR Forward Primer (5′ to 3′)
mmu-miR-151-3p	CTCAACTGGTGTCGTGGAGTCGGCAATTCAGTTGAG CCTCAAGG	ACACTCCAGCTGGGCTAGACTGAGGCTCC
mmu-miR-199a-3p	CTCAACTGGTGTCGTGGAGTCGGCAATTCAGTTGAG TAACCAAT	ACACTCCAGCTGGGACAGTAGTCTGCACAT
mmu-miR-99b-5p	CTCAACTGGTGTCGTGGAGTCGGCAATTCAGTTGAG CGCAAGGT	ACACTCCAGCTGGGCACCCGTAGAACCGAC
mmu-miR-125b-2-3p	CTCAACTGGTGTCGTGGAGTCGGCAATTCAGTTGAG AGGTCCCA	ACACTCCAGCTGGGACAAGTCAGGTTCTTG
mmu-miR-129-5p	CTCAACTGGTGTCGTGGAGTCGGCAATTCAGTTGAG GCAAGCCC	ACACTCCAGCTGGGCTTTTTGCGGTCTGG
mmu-miR-132-5p	CTCAACTGGTGTCGTGGAGTCGGCAATTCAGTTGAG GTAACAAT	ACACTCCAGCTGGGAACCGTGGCTTTCGAT
mmu-miR-196b-5p	CTCAACTGGTGTCGTGGAGTCGGCAATTCAGTTGAG CCCAACAA	ACACTCCAGCTGGGTAGGTAGTTTCCTGTT
mmu-miR-212-3p	CTCAACTGGTGTCGTGGAGTCGGCAATTCAGTTGAG TGGCCGTG	ACACTCCAGCTGGGTAACAGTCTCCAGTCA
mmu-miR-3109-3p	CTCAACTGGTGTCGTGGAGTCGGCAATTCAGTTGAG TATCTGGA	ACACTCCAGCTGGGTAGGGCCATCTCATC
mmu-miR-32-5p	CTCAACTGGTGTCGTGGAGTCGGCAATTCAGTTGAG TGCAACTT	ACACTCCAGCTGGGTATTGCACATTACTAA
mmu-miR-324-3p	CTCAACTGGTGTCGTGGAGTCGGCAATTCAGTTGAG AGCAGCAC	ACACTCCAGCTGGGCCACTGCCCCAGGT
mmu-miR-369-5p	CTCAACTGGTGTCGTGGAGTCGGCAATTCAGTTGAG GCGAATAT	ACACTCCAGCTGGGAGATCGACCGTGTTAT
Universal Reverse primer for qPCR (URP)		TGGTGTCGTGGAGTCG
U6 F		CTCGCTTCGGCAGCACA
U6 R	AACGCTTCACGAATTTGCGT	AACGCTTCACGAATTTGCGT

## Supplementary Materials

Supplementary materials can be found at https://www.mdpi.com/1422-0067/20/21/5506/s1.

## Abbreviations

DCs	Dendritic cells
DEmiR	Differential exosomal miRNA
APCs	Antigen-presenting cells
PPI	Protein–protein interaction network
KDA	Key driver analysis
GO	Gene ontology
KEGG	Kyoto Encyclopedia of Genes and Genomes
MOI	Multiplicity of infection
TEM	Transmission electron microscope
NTA	Nanoparticle tracking analysis
FBS	Fetal bovine serum
PVDF	Polyvinylidene fluoride
